# Exploring sex disparities in cardiovascular disease risk factors using principal component analysis and latent class analysis techniques

**DOI:** 10.1186/s12911-023-02179-3

**Published:** 2023-05-25

**Authors:** Gamal Saad Mohamed Khamis, Sultan Munadi Alanazi

**Affiliations:** Northern Border University (https://www.nbu.edu.sa/en), Arar, Saudi Arabia

**Keywords:** Principal component analysis (PCA), Latent class analysis (LCA), Bayesian information criteria (BIC), Cardiovascular disease (CVD), And risk factors

## Abstract

**Background:**

This study used machine learning techniques to evaluate cardiovascular disease risk factors (CVD) and the relationship between sex and these risk factors. The objective was pursued in the context of CVD being a major global cause of death and the need for accurate identification of risk factors for timely diagnosis and improved patient outcomes. The researchers conducted a literature review to address previous studies' limitations in using machine learning to assess CVD risk factors.

**Methods:**

This study analyzed data from 1024 patients to identify the significant CVD risk factors based on sex. The data comprising 13 features, such as demographic, lifestyle, and clinical factors, were obtained from the UCI repository and preprocessed to eliminate missing information. The analysis was performed using principal component analysis (PCA) and latent class analysis (LCA) to determine the major CVD risk factors and to identify any homogeneous subgroups between male and female patients. Data analysis was performed using XLSTAT Software. This software provides a comprehensive suite of tools for Data Analysis, Machine Learning, and Statistical Solutions for MS Excel.

**Results:**

This study showed significant sex differences in CVD risk factors. 8 out of 13 risk factors affecting male and female patients found that males and females share 4 of the eight risk factors.

Identified latent profiles of CVD patients, suggesting the presence of subgroups among CVD patients. These findings provide valuable insights into the impact of sex differences on CVD risk factors. Moreover, they have important implications for healthcare professionals, who can use this information to develop individualized prevention and treatment plans. The results highlight the need for further research to elucidate these disparities better and develop more effective CVD prevention measures.

**Conclusions:**

The study explored the sex differences in the CVD risk factors and the presence of subgroups among CVD patients using ML techniques. The results revealed sex-specific differences in risk factors and the existence of subgroups among CVD patients, thus providing essential insights for personalized prevention and treatment plans. Hence, further research is necessary to understand these disparities better and improve CVD prevention.

## Background

Cardiovascular disease (CVD) is a group of disorders affecting the heart and blood vessels, including coronary artery disease, heart failure, and arrhythmias. CVD is a leading cause of death worldwide, with an estimated 17.9 million deaths in 2019 [1].

CVD can result from various risk factors, including high blood pressure, high cholesterol, smoking, diabetes, obesity, a family history of CVD, and a sedentary lifestyle. These risk factors can damage the blood vessels, leading to plaque buildup and ultimately resulting in heart disease [2]. Early detection and prognosis of CVD are crucial in reducing mortality rates. CVD can be diagnosed using various methods, including electrocardiogram (ECG), echocardiogram, stress tests, and cardiac catheterization. Additionally, medical history and physical examination can provide valuable information in diagnosing and managing CVD [2].

Adopting a healthy lifestyle can significantly reduce the risk of developing CVD. This includes regular physical activity, maintaining a healthy weight, avoiding smoking, and eating a healthy diet. Moreover, there are significant cardiovascular physiological differences between men and women, and recognizing these differences is essential in preventing and treating CVD [3].

For example, women tend to develop CVD later in life compared to men, and the symptoms of CVD in women can differ from those in men [3]. CVD is a significant global health challenge affecting millions worldwide. The early detection and management of CVD are critical in reducing mortality rates. Adopting a healthy lifestyle and recognizing the physiological differences between men and women are essential in preventing and treating CVD.

The main objectives of this paper are as follows:

- To examine the influence of sex on CVD risk factors by exploring sex differences in cardiovascular risk factors using advanced statistical techniques, such as Principal Component Analysis (PCA) and Latent Class Analysis (LCA).
- To provide a deeper understanding of the underlying factors contributing to an individual's CVD risk profile and to offer insights into sex-specific risk profiles.
- To advance the field of CVD risk factor analysis and to highlight the importance of ongoing research in this area.

The paper is structured as follows: The background section provides a comprehensive literature review of studies that have employed ML methodologies to assess CVD risk factors and analyze their limitations. Furthermore, the method section outlines the dataset and the proposed model, which involves preprocessing and applying PCA and LCA techniques to identify the most significant risk factors contributing to CVD in both men and women. The results section carefully examines the findings to determine the presence of sex-specific risk profiles and assess the significance of any differences found. The discussion section explores the implications of these results. The study is finally concluded in the conclusion section.

A review of the previous related work is presented to gain knowledge of previous research on CVD risk factor analysis tools. In some of the surveyed papers, traditional models were applied to detect the CVD risk factors, whereas, in some other papers, ML algorithms were implemented.

The atherosclerotic cardiovascular disease (ASCVD) Risk-Estimator Plus is a program that evaluates numerous characteristics, such as blood pressure, cholesterol, medical history, and sex, to determine the CVD risk factors. This program can estimate the severity of the abovementioned factors and their changes over time, increasing the risk of heart disease [4]. In [5], The existing sex differences in CVD burden and variables that may facilitate or impede quality CVD preventive care in women were examined. The latent classes of sleep quality based on the Pittsburgh Sleep Quality Index were investigated and analyzed in Chinese adults to discover if they differed between men and women [6].

In [7], it was concluded that the use of nontraditional risk factors in the CVD assessment, such as the ankle-brachial index, the high-sensitivity C-reactive protein (hs-CRP) level, and the coronary artery calcium score together with the Framingham Risk Score model [8], the Pooled Cohort Equations [9] and other models, leads to improved measurements of calibration, discrimination, and risk reclassification.

In [10], three ML classifiers were applied and compared against an assessment tool for CVD risk prediction and against actual CVD patients. The results showed that ML performs comparably well against traditional risk assessment tools in identifying a potential CVD development in an individual.

In [11], the Auto Prognosis model was proposed to improve the accuracy of CVD risk prediction. It was concluded that the information gain achieved by considering more risk factors in the predictive model was significantly higher than the modeling gain achieved by adopting complex predictive models.

In [12], the performance of seven cardiovascular (CV) risk algorithms was evaluated in multi-centric cohort analysis of ankylosing spondylitis (AS) patients. The performance and calibration of traditional CV predictors were compared with the novel ML paradigm. The results showed that the ML algorithms could improve the cardiovascular assessment in patients with AS and demonstrated that the hs-CRP level could be the key feature of an increased risk in these patients.

Shah et al. [13] proposed a selection technique based on the probabilistic PCA (PPCA) of the probabilistic electronic medical record. PPCA’s essential function was used to obtain the most significant predictive characteristics for heart disease prediction.

LCA and the latent transition analysis were applied to investigate the diagnostic accuracy and status change of Alzheimer’s disease (AD) patients [14]. LCA was found to produce more accurate results for classifying and identifying the progression of AD compared to conventional clinical cutoff measures.

Thus, a novel method for diagnosing myocarditis using a combination of Convolutional Neural Network (CNN) and K-Means Clustering (KCL) was proposed [15]. This method first utilizes CNN to extract features from medical images and subsequently employs KCL to classify these features. The evaluation results show that this method outperforms the existing methods.

In [16], the Classification and Regression Tree (CART) algorithm used to predict heart disease and identify the most important features influencing the disease. The model achieved 87% accuracy, indicating its reliability, and the extracted decision rules can simplify the use of clinical purposes.

Furthermore, Random Forest (RF) was utilized to evaluate the risk factors of CVD in Chin [17]. The Random Forest algorithm was found to be the most effective in predicting CVD risk, with an AUC of 0.787.

According to a review of previous related study, various models and techniques have been used to analyze and identify the risk factors associated with CVD. These techniques range from conventional models such as models incorporating the Framingham Risk Score and Pooled Cohort Equations and machine learning algorithms such as CNN and RF.

Four machine learning-based CVD classifiers (RF, SVM, Multi-Layer Perceptron, and Light Gradient Boosting) were developed based on the Korea National Health and Nutrition Survey [18]. The proposed classifiers performed excellently with AUCs greater than 0.853. Thus, using Shapley score-based risk factor analysis, the study identified that the most significant CVD risk factors were age, sex, and prevalence of hypertension. Age, hypertension, and BMI were positively associated with CVD, and sex (female), alcohol consumption, and monthly income were negatively associated. The results showed that feature selection and class balancing techniques effectively improved the interpretability of the models.

Based on the review of the previous related studies, various models and techniques have been implemented to analyze and identify the risk factors associated with CVD. These techniques range from traditional models such as the Framingham Risk Score and Pooled Cohort Equations to machine learning algorithms such as CNN and RF.

The proposed method in this study incorporates both PCA and LCA to conduct a comprehensive evaluation of the relationship between sex and cardiovascular risk factors. This method provides a significant contribution to the field, offering a deeper insight into the complex interplay between sex and cardiovascular risk. Furthermore, the authors also provide a detailed outline of the methodology and results, including any limitations and implications for future research.

## Method

**Fig. 1:**
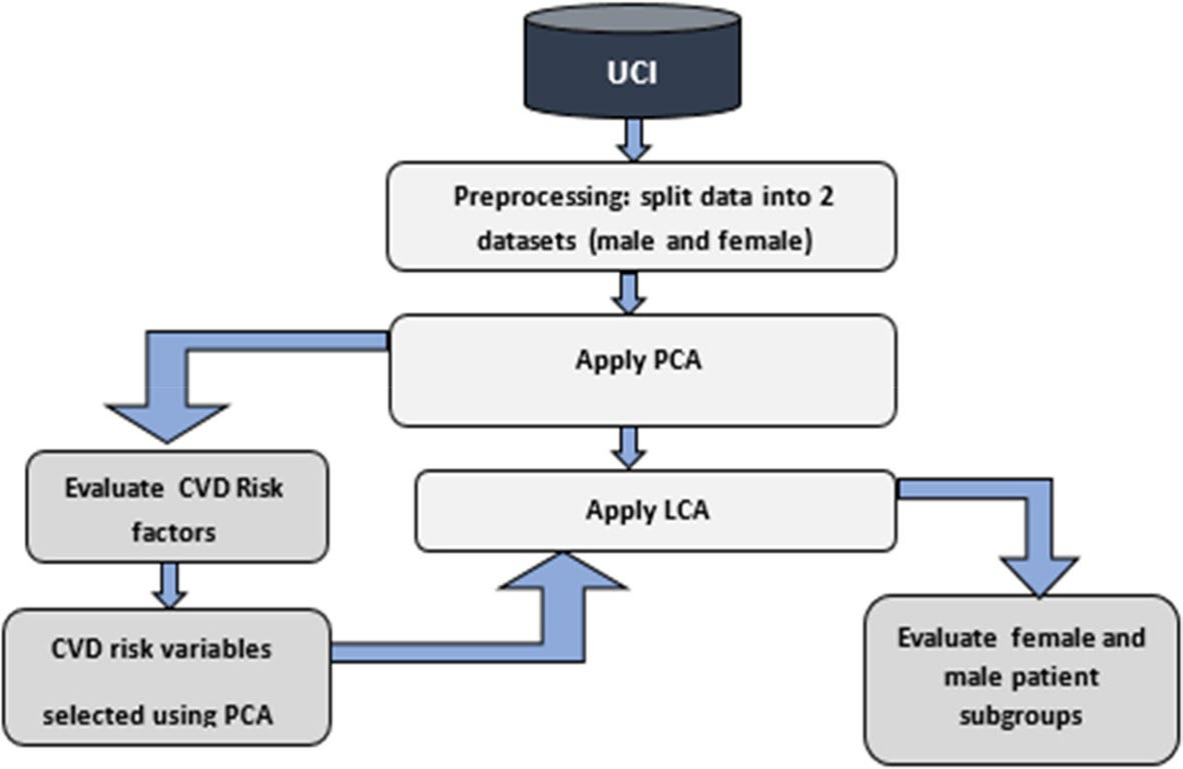
The architecture of the proposed model

The proposed model shown in Fig. 1, which uses a combination of PCA and LCA to assess CVD risk factors and improve CVD diagnosis.

The Cleveland heart dataset obtained from UCI [19] was utilized for this purpose and contained 13 input features, as shown in Table 1.

**Table 1:** Cleveland heart disease dataset features

No	Feature	Feature Discretion	Value Range
1	age	Years	[29,77]
2	sex	Sex	0 = female 1 = male
3	cp	Chest pain	0 = typical angina 1 = atypical angina 2 = non-angina pain 3 = asymptomatic
4	trestbps	Resting blood pressure in mmHg on admission to the hospital	[94, 200]
5	chol	Serum cholesterol in mg/dl	[126, 564]
6	fbs	Fasting blood sugar > 120 mg/dl	0 = false 1 = true
7	restecg	Resting electrocardiographic results	0 = normal 1 = ST-T wave abnormality 2 = left ventricular hypertrophy
8	thalach	Maximum heart rate achieved	[71, 202]
9	exang	Exercise-induced angina	0 = no 1 = yes
10	oldpeak	ST depression induced by exercise relative to rest	[0, 6.2]
11	slope	Slope of the peak exercise ST segment	0 = up sloping 1 = flat 2 = down sloping
12	ca	Number of major vessels colored by fluoroscopy	0 – 3
13	thal	Thallium stress test	1 = normal 2 = fixed defect 3 = reversible defect

### Preprocessing

First, the raw Cleveland samples are fed to the XLSTAT [20] data preparation tool. After preprocessing, all samples with null values are removed, and the dataset is divided into two parts; data associated with female patients and data associated with male patients.

### Assessing CVD factors using Principal Component Analysis (PCA)

PCA is primarily used as a dimensionality reduction approach with data visualization and feature extraction. The standard context for PCA as an exploratory data analysis tool involves a dataset with observations of numerical variables for each of n entities or individuals [21]. These data values define *p n*-dimensional vectors *x*_1_, …, *x*_p_, or, equivalently, an *n*×*p* data matrix *X*, whose *j*_th_ column is the vector *x*_j_ of observations on the *j*_th_ variable. We look for a linear combination of the columns of matrix *X* with the maximum variance. Such linear combinations are given as follows:

$$Xa= \sum_{j=1}^{p}(aj.xj)$$

(where *a* is a vector of constants *a*_1_, *a*_2_, …, *a*_p_ [13]) and provide:

- Quick visualization and analysis of correlations between the *N* variables.
- Visualization and analysis of the M observations (initially described by the *N* variables) on a low-dimension map (the optimal view for a variability criterion).
- Building a set of *P* uncorrelated factors.

**Latent Class Analysis** (**LCA):**

LCA is a cluster-analysis statistical method used for identifying and creating structures from unobserved or latent subgroups, which typically rely on individual responses from multivariate data [22, 23]. LCA can also be utilized as a data reduction tool when analyzing multivariate data [24, 25].

The mathematical model of LCA can be described as follows:

Let *y*_j_ represent the element *j* of a response pattern *y*. Thus, let us establish an indicator function *I* (*y*_j_ = *r*_j_) that equals 1 when the response to variable *j* equals *r*_j_, and equals 0, otherwise. Accordingly, the probability of observing a particular vector of responses is

$$P\left(Y=y\right)=\sum_{c=1}^{c}\gamma c \prod_{j=1}^{j}\prod_{rj=1}^{Rj}{P}_{j.rj\backslash c}^{I(yi=rj)}$$

Here, *Y*_*c*_ is the probability of membership in the latent class *c* and ${P}_{j.rj\backslash c}^{I(yi=rj)}$is the probability of response *r*_j_ to item *j* conditional on the latent class *c* membership. Furthermore, the γ parameters represent a vector of latent class membership probabilities that sum to one, and the ρ parameters represent a matrix of item-response probabilities conditional on the latent class membership. In this analysis, the underlying latent class is CVD patient patterns.

## Results

First, we look at the correlation matrix. This matrix appears in Tables 2 and 3, which illustrate the degree of correlation among CVD risk factors for women and men, respectively.

**Table 2:** Correlation matrix for data associated with female patients

Correlation matrix (Pearson (n)):												
cp	thal	ca	slope	exang	fbs	restecg	oldpeak	chol	trestbps	thalach	age	Variables
-0.034	**0.140**	**0.396**	-0.068	0.016	**0.122**	-0.023	**0.178**	**0.245**	**0.285**	**-0.399**	**1**	age
**0.125**	**-0.153**	**-0.150**	**0.334**	**-0.132**	**-0.158**	-0.077	**-0.249**	0.031	-0.111	**1**	**-0.399**	thalach
**-0.170**	**0.205**	**0.299**	**-0.269**	**0.336**	**0.256**	-0.028	**0.407**	**0.156**	**1**	-0.111	**0.285**	trestbps
-0.058	**0.264**	0.108	**0.122**	0.097	**0.142**	**-0.275**	**0.134**	**1**	**0.156**	0.031	**0.245**	chol
**-0.293**	**0.373**	**0.461**	**-0.598**	**0.192**	**0.128**	-0.086	**1**	**0.134**	**0.407**	**-0.249**	**0.178**	oldpeak
0.064	0.004	**-0.139**	0.083	0.034	**-0.186**	**1**	-0.086	**-0.275**	-0.028	-0.077	-0.023	restecg
-0.003	0.046	**0.279**	0.028	**0.200**	**1**	**-0.186**	**0.128**	**0.142**	**0.256**	**-0.158**	**0.122**	fbs
**-0.438**	**0.248**	0.043	**-0.284**	**1**	**0.200**	0.034	**0.192**	0.097	**0.336**	**-0.132**	0.016	exang
**0.291**	**-0.333**	**-0.219**	**1**	**-0.284**	0.028	0.083	**-0.598**	**0.122**	**-0.269**	**0.334**	-0.068	slope
**-0.266**	**0.287**	**1**	**-0.219**	0.043	**0.279**	**-0.139**	**0.461**	0.108	**0.299**	**-0.150**	**0.396**	ca
**-0.259**	**1**	**0.287**	**-0.333**	**0.248**	0.046	0.004	**0.373**	**0.264**	**0.205**	**-0.153**	**0.140**	thal
**1**	**-0.259**	**-0.266**	**0.291**	**-0.438**	-0.003	0.064	**-0.293**	-0.058	**-0.170**	**0.125**	-0.034	cp

*Values in bold are different from 0 with a significance level of alpha* = *0.05*

**Table 3:** Correlation matrix for data associated with male patients

Correlation matrix (Pearson (n)):												
cp	thal	ca	slope	exang	fbs	restecg	oldpeak	chol	trestbps	thalach	age	Variables
**-0.095**	**0.084**	**0.246**	**-0.219**	**0.140**	**0.126**	**-0.198**	**0.237**	**0.181**	**0.255**	**-0.401**	**1**	age
**0.366**	**-0.075**	**-0.220**	**0.416**	**-0.456**	0.044	**0.095**	**-0.383**	-0.069	-0.015	**1**	**-0.401**	thalach
**0.134**	0.035	0.037	-0.052	-0.046	**0.151**	**-0.187**	**0.091**	**0.084**	**1**	-0.015	**0.255**	trestbps
**-0.116**	**0.107**	**0.100**	**-0.108**	**0.103**	-0.033	**-0.083**	0.055	**1**	**0.084**	-0.069	**0.181**	chol
**-0.125**	**0.141**	**0.128**	**-0.566**	**0.344**	-0.039	-0.027	**1**	0.055	**0.091**	**-0.383**	**0.237**	oldpeak
0.032	-0.015	-0.047	**0.086**	**-0.098**	-0.066	**1**	-0.027	**-0.083**	**-0.187**	**0.095**	**-0.198**	restecg
**0.112**	**-0.076**	**0.086**	**-0.096**	-0.009	**1**	-0.066	-0.039	-0.033	**0.151**	0.044	**0.126**	fbs
**-0.387**	**0.157**	**0.110**	**-0.260**	**1**	-0.009	**-0.098**	**0.344**	**0.103**	-0.046	**-0.456**	**0.140**	exang
0.070	-0.024	-0.020	**1**	**-0.260**	**-0.096**	**0.086**	**-0.566**	**-0.108**	-0.052	**0.416**	**-0.219**	slope
**-0.142**	**0.092**	**1**	-0.020	**0.110**	**0.086**	-0.047	**0.128**	**0.100**	0.037	**-0.220**	**0.246**	ca
**-0.133**	**1**	**0.092**	-0.024	**0.157**	**-0.076**	-0.015	**0.141**	**0.107**	0.035	**-0.075**	**0.084**	thal
**1**	**-0.133**	**-0.142**	0.070	**-0.387**	**0.112**	0.032	**-0.125**	**-0.116**	**0.134**	**0.366**	**-0.095**	cp

*Values in bold are different from 0 with a significance level of alpha* = *0.05*

By comparing the correlation between risk factors for women and that for men, no significant difference in the degree of correlation between age and other variables associated with both male and female patients is observed.

The age variable is positively correlated with variables *trestbps, chol, old peak, fbs, exang, ca,* and *thal* for both female and male patients. Also, the correlation matrix shown in Tables 2 and 3 indicates that the risk variables that are positively correlated for female patients are negatively correlated for male patients. For example, *chol* and *thalac* are positively correlated for female patients, whereas they are negatively correlated for male patients. The same is true for variables *chol* and *fbs*.

The above findings for the risk variables associated with female and male patients were extracted from the correlation matrix. These findings indicate a set of risk variables that affect female patients. In this set, the variables are positively correlated with each other. The corresponding set of risk variables that affect male patients is somewhat different.

The same findings can be extracted from Tables 4 and 5, which show the squared cosine of the variables for both datasets. The squared cosine analysis is used to avoid interpretation errors due to projection effects. If the squared cosine of a variable associated with a factor Fi is low, the variable should not be interpreted using factor Fi. In Tables 4 and 5, the values in bold associated with each variable correspond to the factor for which the squared cosine is the largest.

**Table 4:** Squared cosine of the variables in the dataset associated with female patients

F5	F4	F3	F2	F1	
0.085	0.004	**0.282**	0.188	0.206	age
0.002	0.028	**0.354**	0.008	0.202	thalach
0.007	0.090	0.002	0.011	**0.375**	trestbps
0.238	0.010	0.128	**0.355**	0.076	chol
0.029	0.097	0.002	0.020	**0.571**	oldpeak
0.155	0.141	0.168	**0.214**	0.025	restecg
0.199	**0.237**	0.005	0.213	0.111	fbs
0.011	**0.305**	0.169	0.089	0.241	exang
0.054	0.077	0.006	0.235	**0.397**	slope
0.029	0.039	0.028	0.077	**0.396**	ca
0.190	0.051	0.025	0.009	**0.334**	thal
0.010	0.003	0.128	0.111	**0.268**	cp

**Table 5:** Squared cosine of the variables in the dataset associated with male patients

F5	F4	F3	F2	F1	
0.003	0.009	0.035	0.196	**0.319**	age
0.008	0.039	0.004	0.017	**0.598**	thalach
0.000	0.064	0.006	**0.470**	0.021	trestbps
0.000	0.158	**0.182**	0.014	0.074	chol
0.041	0.047	0.190	0.001	**0.458**	oldpeak
**0.447**	0.006	0.048	0.165	0.054	restecg
0.020	0.216	0.017	**0.280**	0.001	fbs
0.038	0.006	0.000	0.101	**0.419**	exang
0.001	0.022	0.314	0.007	**0.397**	slope
0.196	**0.201**	0.175	0.014	0.124	ca
0.164	**0.228**	0.138	0.021	0.065	thal
0.051	0.042	0.118	**0.235**	0.218	cp

In Table 4, which represents female patients’ data, factors F1 and F2 are the two best factors describing the data. They include 8 variables (*trestbps, chol, oldpeak, restecg, slope, ca, thal,* and *cp*) interpreted by them.

In Table 5, which represents male patients' data, factors F1 and F2 include 8 variables (*age, thalach, trestbps, oldpeak, fbs, exang, slope,* and *cp*) interpreted by them.

The values of the squared cosine of the variables associated with female and male patients indicate that, from 8 variables interpreted by factors associated with female patients and 8 variables interpreted by factors associated with male patients, there are 4 shared variables. The other 4 variables are different.

**Fig. 2:**
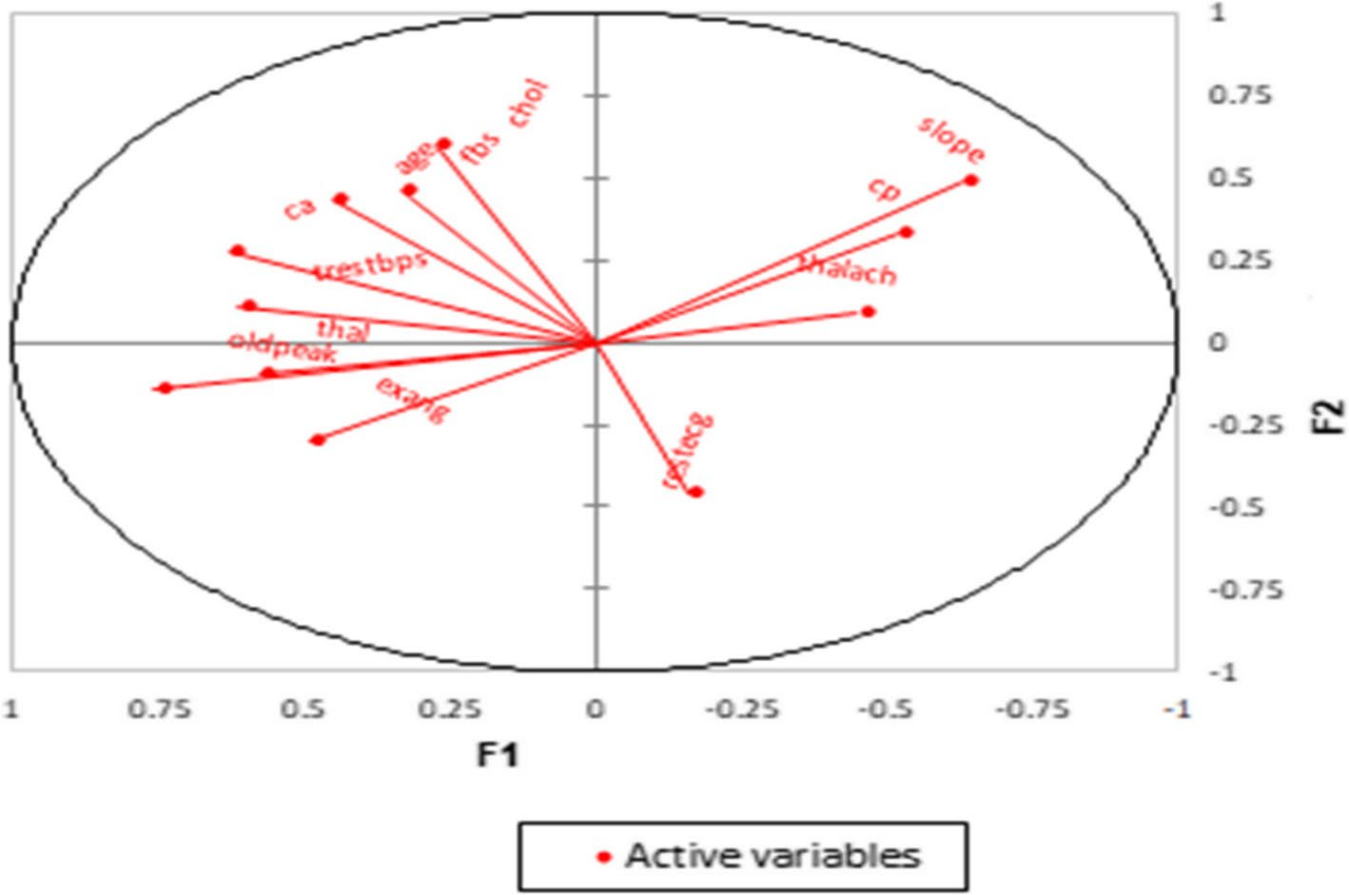
Correlation circle for data associated with female patients

**Fig. 3:**
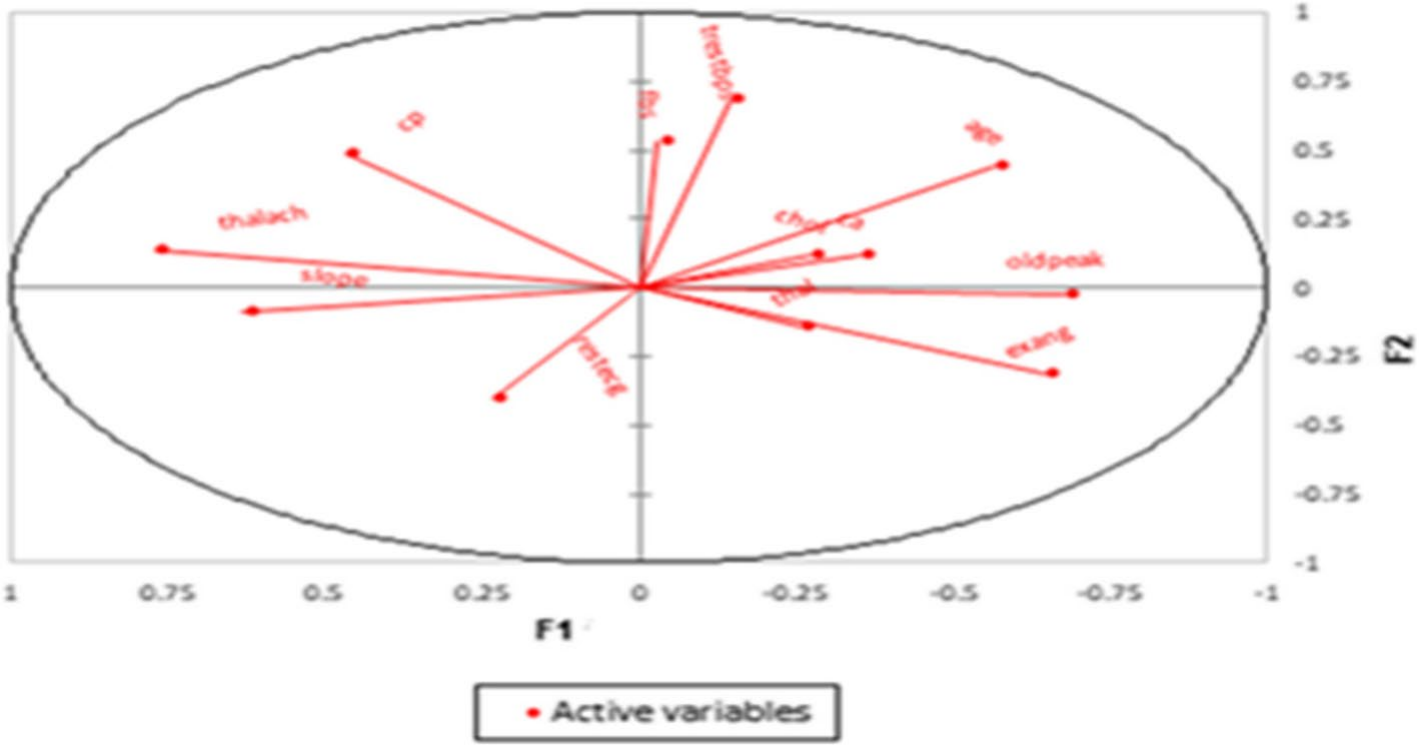
Correlation circle for data associated with male patients

Figures 2 and 3 illustrate the correlation circles. The first most explanatory dimension regarding the variance is called F1 and is plotted on the horizontal axis. The second-most explanatory dimension is called F2 and is plotted on the vertical axis. Inside this 2-dimensional circle, the original 13 variables are projected in red onto this 2-dimensional factor space. The smaller the angle between two lines, the higher the correlation between the two corresponding variables is. If two lines are orthogonal to each other (at a 90-degree angle), they are uncorrelated. If two lines point in opposite directions, they are negatively correlated.

**Fig. 4:**
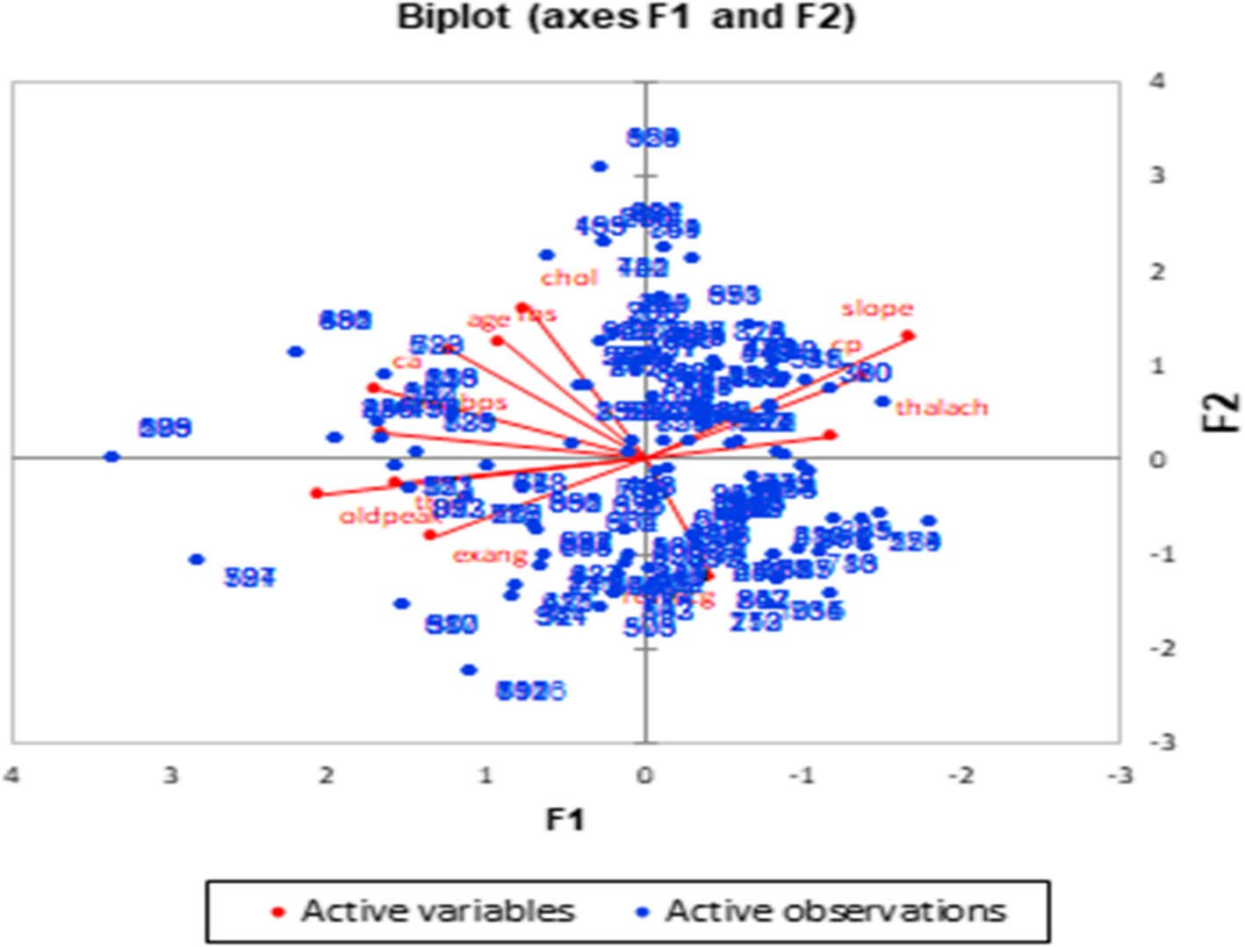
Distribution of female patients, according to the risk factors affecting them

**Fig. 5:**
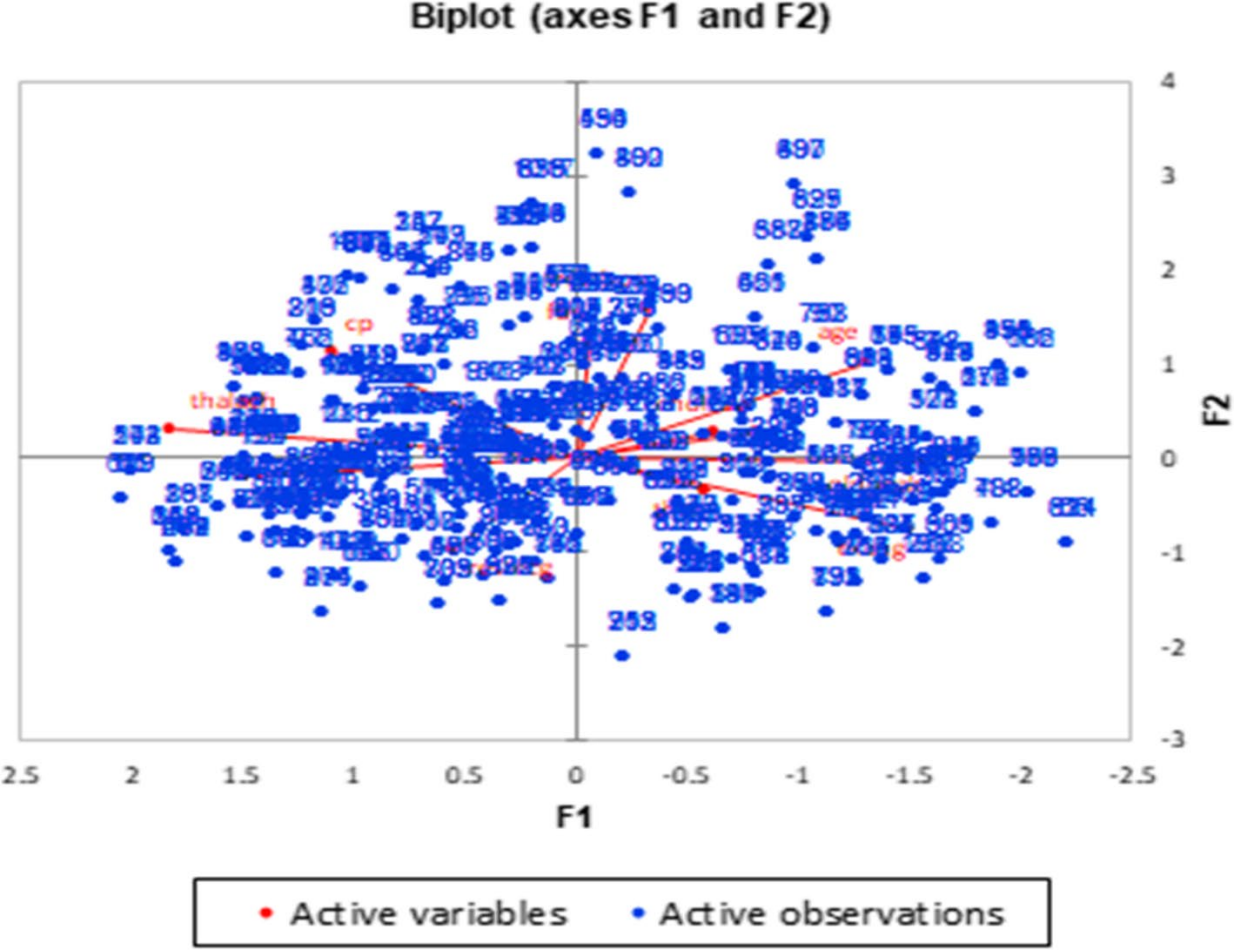
Distribution of male patients, according to the risk factors affecting them

The PCA biplots showed in Figs. 4 and 5 illustrate the correlation circle of the active variables and the scatter plot of the active observations. In these figures, F1 is the first factor or principal component 1, and F2 is the second factor or principal component 2.

Previously, the risk factors for CVD patients were assessed on the basis of a patient’s sex using the PCA method. It was found that some risk factors associated with male patients were positively correlated with each other, whereas other risk factors were negatively correlated with each other. Also, some risk factors associated with female patients that were positively correlated with each other were different from the corresponding risk factors associated with male patients. These results are presented in Tables 4 and 5.

In addition, from the 8 out of 13 risk factors that affect male and female patients, it was found that males and females share 4 of the 8 risk factors.

To answer the question, "are there any latent subgroups within each group of patients (females and males)?” LCA was applied to the female and male patients’ datasets to extract the (latent) subgroups within each group.

The PCA results showed that eight risk variables were associated with both male and female patients, with four of these variables being shared by both groups. The analysis revealed that eight variables impacted each patient group, including male and female patients.

These eight factors are the LCA inputs, which were used to detect and analyze the subgroups of each type.

Tables 6 and 7 present characteristics and responses to CVD risk variables. It can be observed that there are 311 patients representing female patients and 713 representing male patients. Eight CVD risk variables associated with female and male patients are also observed. The data types of these variables range from quantitative data types to qualitative data types.

**Table 6:** Characteristics of data associated with the female patient (Number of patients N = 311)

Feature Discretion	Value Range
cp: Chest pain	0 = typical angina 133 (42.7%) 1 = atypical angina 57 (18.3%) 2 = non-angina pain 109 (35%) 3 = asymptomatic 13 (4%)
trestbps: Resting blood pressure in mmHg on admission to the hospital	Min: 94 Mean: 133.7019 Max: 200
chol: Serum cholesterol in mg/dl	Min: 126 Mean: 261.4551 Max: 564
restecg: Resting electrocardiographic results	0 = normal 144 (46.3%) 1 = ST-T wave abnormality 156 (50.2%) 2 = left ventricular hypertrophy 11 (3.5%)
oldpeak: ST depression induced by exercise relative to rest	Min: 0 Mean: 0.921154 Max: 6.2
slope: The slope of the peak exercise ST segment	0 = up sloping 17 (5.5%) 1 = flat 149 (47.9%) 2 = down sloping 145 (46.6%)
ca: Number of significant vessels colored by fluoroscopy	0 – 204 (65.5%) 1 – 49 (15.8%) 2 – 45 (14.5%) 3 – 13 (4.2%)
thal: Thallium stress test	1 = normal 7 (2.3%) 2 = fixed defect 250 (80.4%) 3 = reversible defect 54 (17.4%)

**Table 7:** Characteristics of data associated with the male patient (Number of patients N = 713)

Feature Discretion	Value Range
age	Min: 29 Mean: 53.8 Max: 77
cp: Chest pain	0 = typical angina 364 (51.1%) 1 = atypical angina 110 (15.4%) 2 = non-angina pain 175 (24.5%) 3 = asymptomatic 64 (9%)
trestbps: Resting blood pressure in mmHg on admission to the hospital	Min: 94 Mean: 130.7 Max: 192
thalach: Maximum heart rate achieved	Min: 71 Mean: 148.4 Max: 202
oldpeak: ST depression induced by exercise relative to rest	Min: 0 Mean: 1.137 Max: 5.6
fbs: Fasting blood sugar s 120 mg/dl	0 = false 602 (84.4%) 1 = true 111 (15.6%)
exang: Exercise-induced angina	0 = no 442 (62%) 1 = yes 271 (38%)
thal: Thallium stress test	1 = normal 64(9%) 2 = fixed defect 292 (41%) 3 = reversible defect 357 (50%)

One of the main objectives when applying LCA is to determine the maximum number of classes (subgroups). However, there is no specific way to do this. To deal with this issue, we relied on the fact that the data contain two main patient categories; the sick patient category and the healthy patient category.

Considering the healthy patient category as the first category, it can be assumed that there are subgroups within the CVD patient category. These subgroups can be divided into three categories; the category of patients in the early stages of the disease, the category of patients with moderate symptoms, and the category of patients in the critical stage. Therefore, a total of four classes can be considered.

The analysis starts with one class. Then, the models are specified by adding one class at a time. The final model selection and its data fitness evaluation can be achieved using statistical criteria. In this paper, Bayesian information criteria (BIC) are used to compare the LCA models; a lower BIC indicates better fitness [26]. Other criteria are also examined, including the Akaike information criteria (AIC); a lower AIC also indicates better fitness.

The results obtained using LCA identifies the latent profiles of CVD patients. These profiles suggest that classes (subgroups) exist among CVD patients. The LCA results for class models are presented in Tables 8 and 9. It can be observed that the BIC and AIC recommend a four-class model for both male and female patients. The BIC is considered the most reliable fit statistic in LCA (and AIC). Consequently, a four-class model for both males and females was selected.

**Table 8:** Evaluation class solutions for female patients

AIC3 (LL)	AIC (LL)	BIC (LL)	LL	No of Clusters
416,002.902	415,963.902	416,300.808	− 207,942.951	2
403,840.912	403,781.912	404,291.590	− 201,831.956	3
**393,483.737**	**393,404.737**	**394,087.187**	− 196,623.368	4

**Table 9:** Evaluation class solutions for male patients

AIC3 (LL)	AIC (LL)	BIC (LL)	LL	No of Clusters
1,288,942.275	1,288,903.275	1,289,276.472	− 644,412.638	2
1,265,633.245	1,265,574.245	1,266,138.825	− 632,728.123	3
**1,250,116.639**	**1,250,037.639**	**1,250,793.601**	− 624,939.819	4

### Female patient subgroups (clusters)

The responses of clusters (subgroups) of female patients to each of the qualitative variables used in building the LCA model are shown in Table 10. The values ​​of the quantitative variables are shown in Table 11.

**Table 10:** Responses of the female patient clusters to each qualitative variable

Cluster 4	Cluster 3	Cluster 2	Cluster 1	Category	Variable
0.084 (22)	0.129 (33)	0.213 (66)	0.574 (191)		(Cluster size)
0.000	0.574	0.043	0.844	1 (0–0.9)	oldpeak
0.000	0.342	0.708	0.156	2 (1–1.9)	
0.305	0.084	0.108	0.000	3 (2–2.9)	
0.160	0.000	0.141	0.000	4 (3–3.9)	
0.399	0.000	0.000	0.000	5 (4–4.9)	
0.046	0.000	0.000	0.000	6 (5–5.9)	
0.091	0.000	0.000	0.000	7 (6–6.9)	
1.000	0.226	0.357	0.486	0	restecg
0.000	0.774	0.457	0.514	1	
0.000	0.000	0.186	0.000	2	
0.524	0.084	0.000	0.018	0	slope
0.476	0.000	1.000	0.401	1	
0.000	0.916	0.000	0.582	2	
0.000	0.595	0.549	0.777	0	ca
0.000	0.000	0.112	0.223	1	
0.692	0.405	0.225	0.000	2	
0.308	0.000	0.114	0.000	3	
0.000	0.000	0.000	0.016	0	thal
0.194	0.000	0.000	0.000	1	
0.160	1.000	0.454	0.957	2	
0.647	0.000	0.546	0.027	3	
1.000	0.144	0.827	0.288	0	cp
0.000	0.500	0.000	0.193	1	
0.000	0.000	0.173	0.519	2	
0.000	0.357	0.000	0.000	3

**Table 11:** Values ​​of the quantitative variables for female Clusters

Variable Cluster	chol	trestbps
1	Min: 141	Min: 94
	Mean: 251	Mean: 128.64
*N* = 191	Max: 417	Max: 180
2	Min: 149	Min: 100
	Mean: 285.7	Mean: 136.4
*N* = 66	Max: 564	Max: 180
3	Min: 195	Min: 106
	Mean: 264.8	Mean: 265.8
*N* = 33	Max: 342	Max: 160
4	Min: 164	Min: 140
	Mean: 273.5	Mean: 162.3
*N* = 22	Max: 407	Max: 200

In this evaluation, the patient’s profile is obtained using each variable and the categories or values in each variable (Table 10). Next, we look at conditional probabilities. For example, in Cluster 1, approximately 84% of patients are likely to exhibit ST depression induced by exercise relative to rest (*oldpeak*, category 1). In Cluster 2, approximately 96% of patients are in the normal range of *oldpeak* (categories 2, 3, and 4). In Cluster 3, approximately 57% of patients are likely to exhibit ST depression induced by exercise relative to rest (*oldpeak*, category 1), and 43% are in the normal range of *oldpeak* (categories 2, 3, and 4). In Cluster 4, approximately 86% of patients are in the normal range of *oldpeak* (categories 2, 3, and 4), and 14% are in the abnormal (elevation) range of *oldpeak* (categories 6 and 7). The electrocardiographic results (*restecg*) indicate that approximately 49% of patients in Cluster 1, 36% in Cluster 2, 23% in Cluster 3, and 100% in Cluster 4 exhibit normal electrocardiographic behaviors (category 0). Also, approximately 51% of patients in Cluster 1, 46% in Cluster 2, and 77% in Cluster 3 exhibit wave abnormality (category 1). Finally, approximately 19% of patients in Cluster 2 suffer from left ventricular hypertrophy (category 2).

The slope of the peak exercise ST segment (*slope*) results indicates that approximately 40% of patients in Cluster 1, 100% in Cluster 2, and 48% in Cluster 4 exhibit a flat slope of the peak exercise ST segment (category 1). Also, 58% of patients in Cluster 1 and 92% in Cluster 3 exhibit a downslope (category 2). The number of major vessels colored by fluoroscopy (*ca*) results indicate that approximately 77% of patients in Cluster 1, 55% in Cluster 2, and 60% in Cluster 3 exhibit no major vessels colored by fluoroscopy (category 0). The chest pain (*cp*) results indicate that 29% of individuals in Cluster 2, 83% in Cluster 2, 14% in Cluster 3, and 100% in Cluster 4 exhibit typical angina (category 0). Also, approximately 19% of patients in Cluster 1 and 50% in Cluster 3 exhibit atypical angina (category 1). Finally, approximately 52% of Cluster 1 and 17% of Cluster 2 exhibit non-angina pain (category 2), and approximately 36% of Cluster 3 are asymptomatic (category 3).

**Fig. 6:**
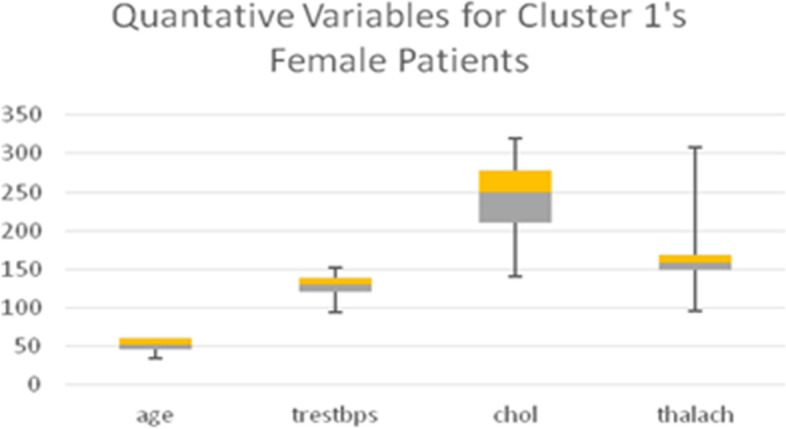
Min, first quartile, mean, third quartile, and max values of quantitative variables for the female patients of Cluster 1

**Fig. 7:**
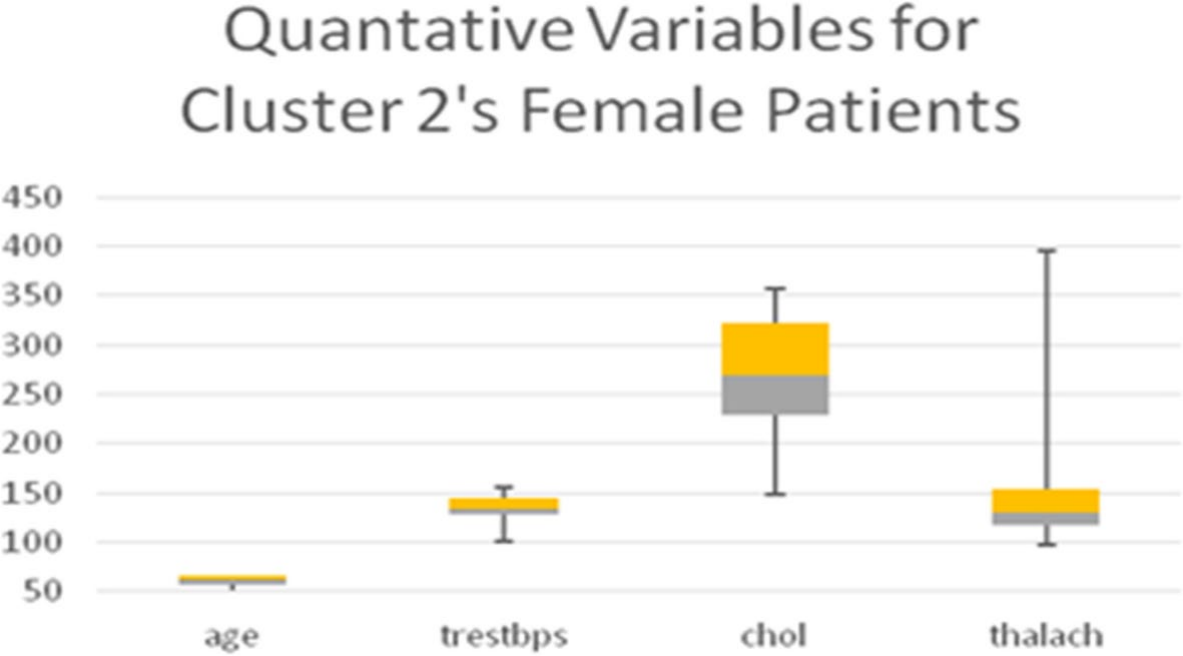
Min, first quartile, mean, third quartile, and max values of quantitative variables for the female patients of Cluster 2

**Fig. 8:**
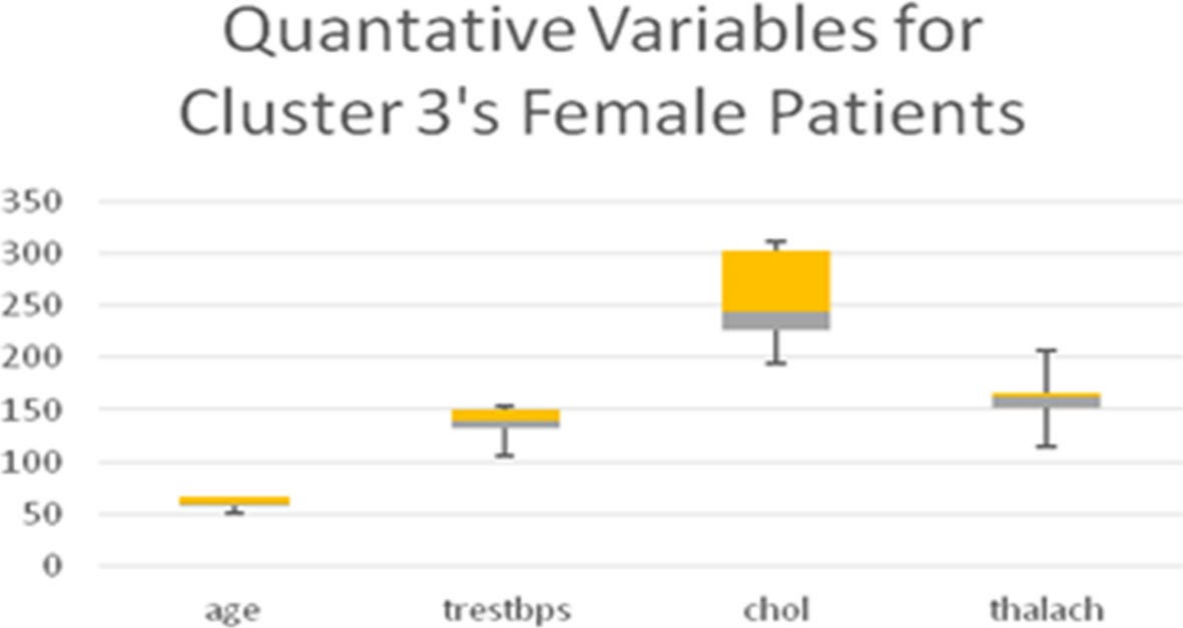
min, first quartile, mean, third quartile, and max values of quantitative variables for the female patients of Cluster 3

**Fig. 9:**
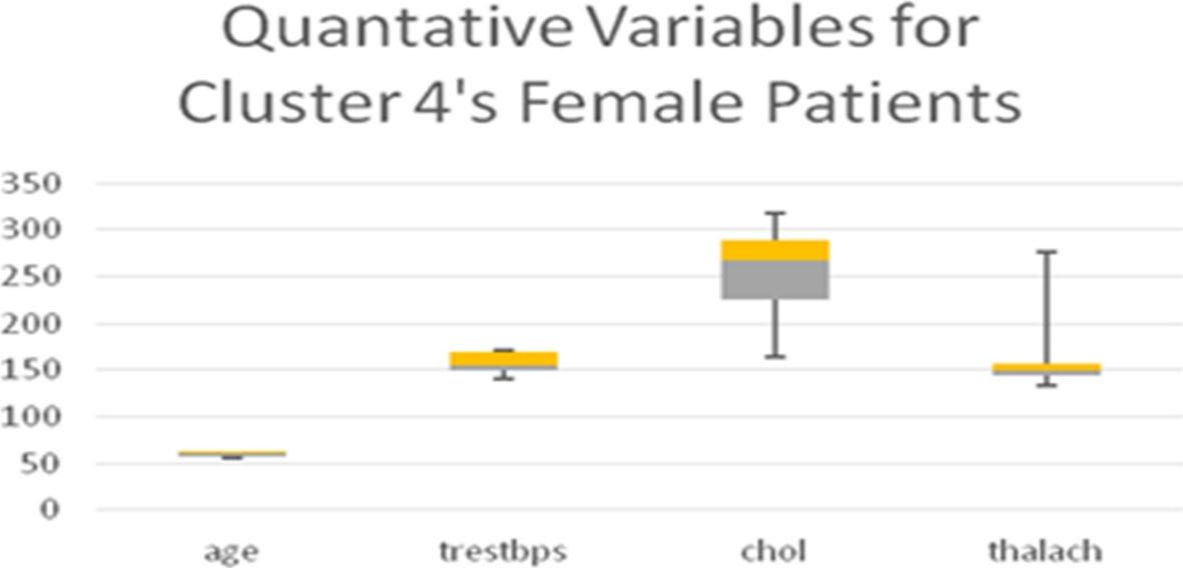
Min, first quartile, mean, third quartile, and max values of quantitative variables for the female patients of Cluster 4

Table 11 shows the quantitative variables cholesterol (*chol*) in mg/dl and blood pressure (*trestbps*), which represent the remainder of the 8 variables used in the LCA model. Boxplots for all the quantitative variables in each Cluster are presented in Figs. 6, 7, 8, and 9 (the min, first quartile, mean, third quartile, and max value of each variable are illustrated).

### Male patient subgroups (clusters):

The responses of clusters (subgroups) of male patients to the CVD risk variables used in building the LCA model. The evaluation of each variable and the categories for each variable are shown in Table 12. The values ​​of the quantitative variables are shown in Table 13.

**Table 12:** Responses of the male patient clusters to each qualitative variable

Cluster 4	Cluster 3	Cluster 2	Cluster 1	Category	Variable
0.187 (141)	0.204 (182)	0.250 (164)	0.359 (226)		(Cluster size)
0.330	0.000	0.650	0.899	1 (0–0.9)	oldpeak
0.325	0.318	0.329	0.078	2 (1–1.9)	
0.222	0.413	0.000	0.000	3 (2–2.9)	
0.101	0.204	0.000	0.024	4 (3–3.9)	
0.022	0.045	0.020	0.000	5 (4–4.9)	
0.000	0.021	0.000	0.000	6 (5–5.9)	
0.737	0.862	0.850	0.879	0	fbs
0.263	0.138	0.150	0.121	1	
0.778	0.262	0.438	0.968	0	exang
0.222	0.738	0.562	0.032	1	
0.148	0.181	0.000	0.036	0	slope
0.852	0.779	0.370	0.048	1	
0.000	0.040	0.630	0.916	2	
0.665	0.359	0.352	0.738	0	ca
0.216	0.310	0.358	0.108	1	
0.029	0.224	0.188	0.062	2	
0.089	0.107	0.086	0.027	3	
0.000	0.000	0.016	0.065	4	
0.000	0.029	0.000	0.000	0	thal
0.157	0.156	0.062	0.000	1	
0.323	0.164	0.216	0.790	2	
0.519	0.651	0.721	0.210	3	
0.001	1.000	0.871	0.148	0	cp
0.124	0.000	0.000	0.404	1	
0.527	0.000	0.082	0.394	2	
0.348	0.000	0.046	0.053	3

**Table 13:** Values ​​of the quantitative variables for male Clusters

Variable Cluster	age	thalach	trestbps
1	Min: 29	Min: 123	Min: 101
	Mean: 49	Mean: 165.7	Mean: 130.4
*N* = 226	Max: 70	Max: 417	Max: 192
2	Min: 35	Min: 71	Min: 94
	Mean: 55.6	Mean: 146.8	Mean: 127.7
*N* = 164	Max: 77	Max: 186	Max: 160
3	Min: 35	Min: 88	Min: 104
	Mean: 55.8	Mean: 126	Mean: 131.2
*N* = 182	Max: 70	Max: 170	Max: 170
4	Min: 37	Min: 103	Min: 100
	Mean: 56	Mean: 151	Mean: 134
*N* = 141	Max: 70	Max: 194	Max: 180

In Cluster 1, approximately 90% of patients are likely to exhibit ST depression induced by exercise relative to rest (*oldpeak*, category 1). In Cluster 2, approximately 65% of patients are also likely to exhibit ST depression (*oldpeak*, category 1), 33% are in the normal range of *oldpeak* (category 2), and 2% are in the abnormal (elevation) range of *oldpeak* (category 5). In Cluster 3, approximately 94% of patients are in the normal range of *oldpeak* (categories 2, 3, and 4). In Cluster 4, approximately 65% of patients are in the expected degree of *oldpeak* (categories 2, 3, and 4), 33% are likely to exhibit ST depression (*oldpeak*, category 1), and 2% are in the abnormal (elevation) range of *oldpeak* (category 6). The fasting blood sugar (*fbs*) results indicate that approximately 88% of patients in Cluster 1, 85% in Cluster 2, 86% in Cluster 3, and 74% in Cluster 4 exhibit *fbs* > 120 mg/dl (category 0). The rest exhibits *fbs* < 120 mg/dl (category 1).

Stable angina is usually triggered by physical activity (*exang*). Approximately 97% of patients in Cluster 1, 44% in Cluster 2, 26% in Cluster 3, and 78% in Custer 4 do not exhibit stable angina related to motor activities and physical exercise (category 0). Also, approximately 3% of patients in Cluster 1, 56% in Cluster 2, 74% in Cluster 3, and 22% in Cluster 4 exhibit stable angina usually triggered by physical activity (category 1).

The slope of the peak exercise ST segment (*slope*) results indicates that approximately 5% of patients in Cluster 1, 37% in Cluster 2, 78% in Cluster 3, and 85% in Cluster 4 exhibit a flat slope of the peak exercise ST segment (category 1). Also, approximately 92% of patients in Cluster 1 and 63% in Cluster 2 exhibit a downward slope of the peak segment (category 2). The number of significant vessels colored by fluoroscopy (*ca*) results indicate that approximately 74% of patients in Cluster 1, 35% in Cluster 2, 36% in Cluster 3, and 66% in Cluster 4 exhibit no major vessels colored by fluoroscopy (category 0). The chest pain (*cp*) results indicate that approximately 15% of patients in Cluster 1, 87% in Cluster 2, and 100% in Cluster 3 exhibit typical angina, defined as a substernal chest pain induced by physical exertion (category 0). Also, approximately 40% of patients in Cluster 1 and 12% in Cluster 4 suffer from atypical angina (category 0). Finally, approximately 40% of patients in Cluster 1 and 53% in Cluster 2 exhibit non-angina pain (category 2).

**Fig. 10:**
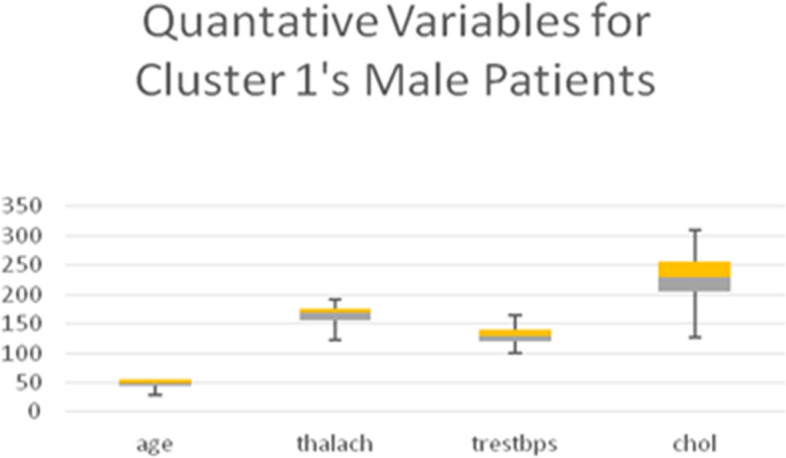
Min, first quartile, mean, third quartile, and max values of quantitative variables for the male patients of Cluster 1

**Fig. 11:**
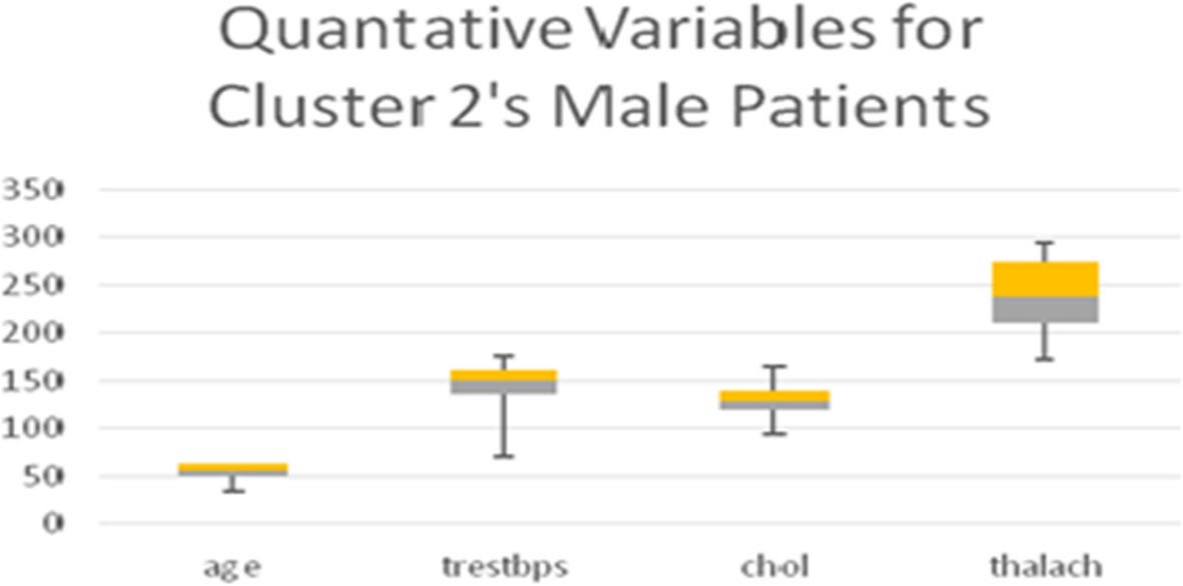
Min, first quartile, mean, third quartile, and max values of quantitative variables for the male patients of Cluster 2

**Fig. 12:**
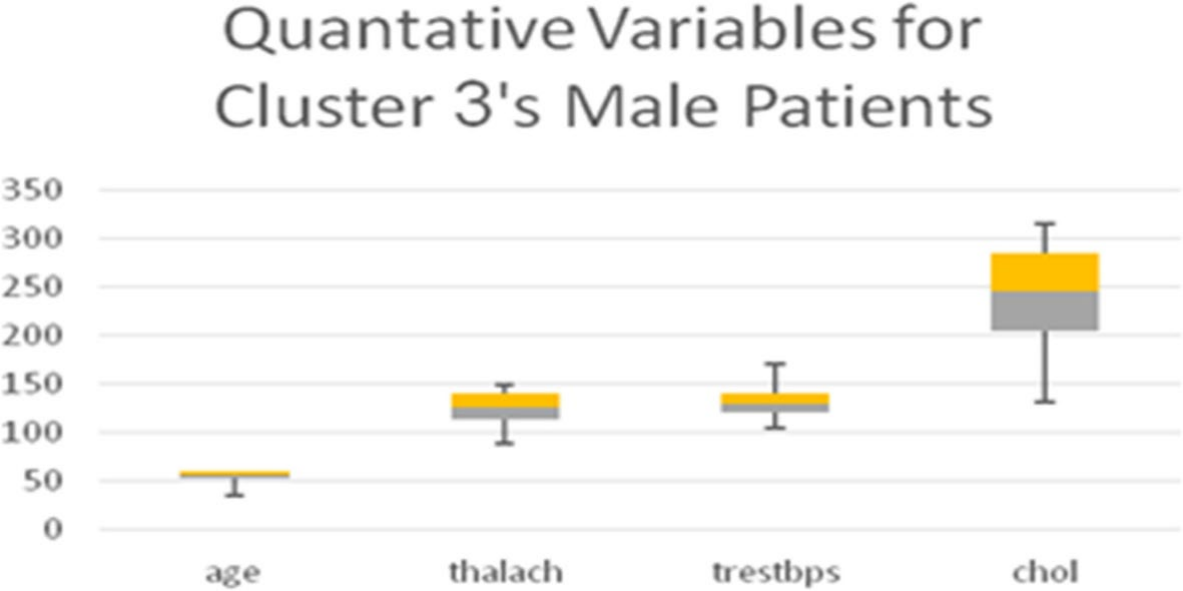
Min, first quartile, mean, third quartile, and max values of quantitative variables for the male patients of Cluster 3

**Fig. 13:**
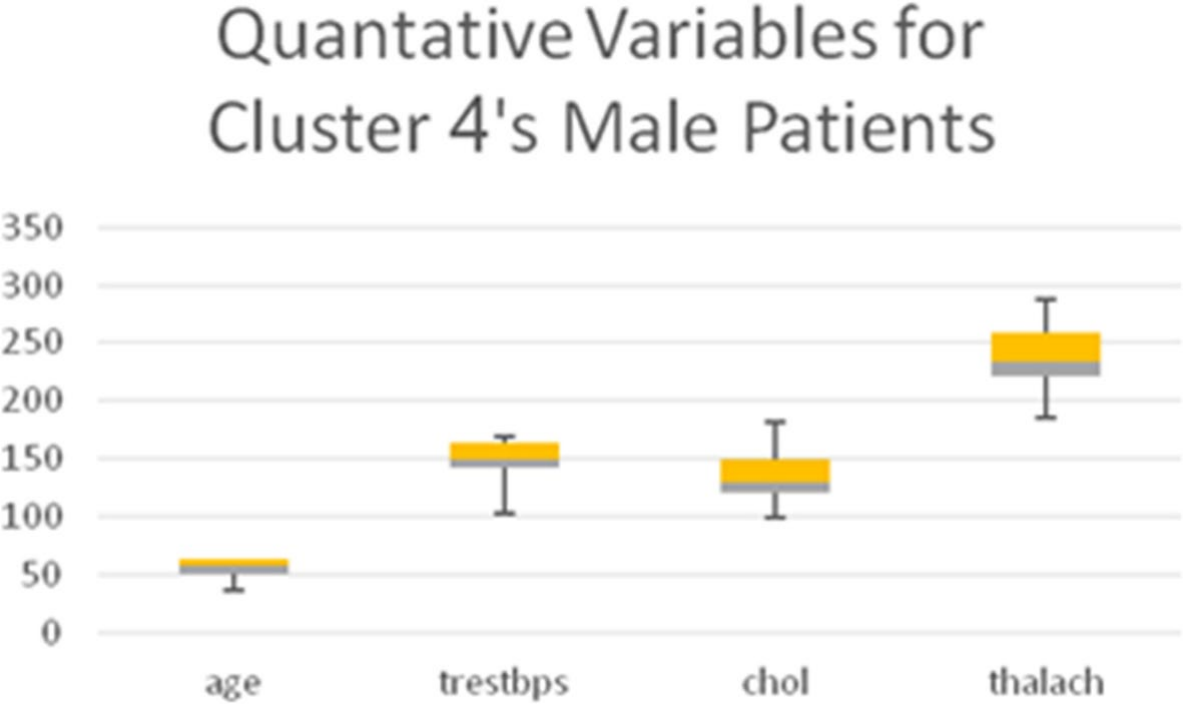
Min, first quartile, mean, third quartile, and max values of quantitative variables for the male patients of Cluster 4

Table 13 shows the quantitative variables *thalach* (maximum heart rate achieved), *age*, and *trestbps* (blood pressure), representing the remainder of the 8 variables used in the LCA model. Boxplots for all quantitative variables in each Cluster are presented in Figs. 10, 11, 12, and 13 (the min, first quartile, mean, third quartile, and max value of each variable are illustrated).

## Discussion

This study explored the relationship between sex and CVD risk factors using ML techniques. The importance of considering sex differences in identifying and managing CVD risk factors was also highlighted.

The study demonstrated sex-specific differences in CVD risk factors, with males and females sharing only four out of the eight risk factors that affect both groups. Furthermore, the finding of the latent profiles of patients with CVD suggests that subgroups among patients with CVD can inform personalized prevention and treatment plans for healthcare professionals.

## Limitation

Expected limitations of this study may include the limited sample size and use of a single data set, which may only partially represent part of the population.

This study provides valuable insights into sex-specific risk profiles, which can inform healthcare professionals in designing prevention and treatment plans for individual patients. However, further research is required to validate these findings and understand the underlying processes that lead to sex differences in CVD risk factors.

Future study should include validating these findings using more extensive and diverse datasets and explore the potential of machine learning models to predict the CVD risk based on sex-specific risk profiles. In this regard, the researchers plan to conduct a future study that uses ML applications on all data sets available in data stores, such as Z-Alizadeh Sani Data Set from UCI and CVD from IEEE DataPort.

## Conclusion

In this work, PCA was used to investigate the most significant CVD risk factors affecting patients based on sex.

The PCA approach results indicated that, for male patients, some risk variables are positively correlated, whereas other risk variables are negatively correlated with each other. Also, the risk variables positively correlated with each other in female patients differ from the corresponding risk variables in male patients. Furthermore, male and female patients share four of the eight risk variables that affect both genders.

Additionally, LCA was used to investigate the existence of any homogenous subgroups between male and female patients. The results of LCA revealed latent profiles of CVD patients, implying that classes (subgroups) exist among CVD patients.

The idea behind examining the presence of subgroups of heart patients is to know the degree of disease progression for each group and identify the subgroup with a greater risk than the rest of the other groups.

BIC and AIC recommended a four-class model for male and female patients. One of the four subgroups contained healthy people, whereas the other three included people with CVD to varying degrees.

Future work will use a dataset from multiple repositories to build a classification model. The model aims to consider the sex differences in CVD risk factors and improve the accuracy of risk assessment for patients.

## Data Availability

The datasets generated and analyzed during the current study are available in the UCI Machine Learning repository https://archive.ics.uci.edu/ml/datasets/heart+disease
